# Determination of Coenzyme A and Acetyl-Coenzyme A in Biological Samples Using HPLC with UV Detection

**DOI:** 10.3390/molecules22091388

**Published:** 2017-08-23

**Authors:** Yevgeniya I. Shurubor, Marilena D’Aurelio, Joanne Clark-Matott, Elena P. Isakova, Yulia I. Deryabina, M. Flint Beal, Arthur J. L. Cooper, Boris F. Krasnikov

**Affiliations:** 1 Feil Family Brain and Mind Research Institute, Weill Cornell Medicine, New York, NY 10065, USA; mad2003@med.cornell.edu (M.D.); fbeal@med.cornell.edu (M.F.B.); 2 Beth Israel Deaconess Medical Center, Harvard Medical School, Boston, MA 02215, USA; jmatott@bidmc.harvard.edu; 3 Bach Institute of Biochemistry, Research Center of Biotechnology of the Russian Academy of Sciences, Moscow 119071, Russia; elen_iss@mail.ru (E.P.I.); yul_der@mail.ru (Y.I.D.); boris_krasnikov@nymc.edu (B.F.K.); 4 Department of Biochemistry and Molecular Biology, New York Medical College, Valhalla, NY 10595, USA

**Keywords:** Acetyl-coenzyme A, coenzyme A, high performance liquid chromatography, UV detection

## Abstract

Coenzyme A (CoA) and acetyl-coenzyme A (acetyl-CoA) play essential roles in cell energy metabolism. Dysregulation of the biosynthesis and functioning of both compounds may contribute to various pathological conditions. We describe here a simple and sensitive HPLC-UV based method for simultaneous determination of CoA and acetyl-CoA in a variety of biological samples, including cells in culture, mouse cortex, and rat plasma, liver, kidney, and brain tissues. The limits of detection for CoA and acetyl-CoA are >10-fold lower than those obtained by previously described HPLC procedures, with coefficients of variation <1% for standard solutions, and 1–3% for deproteinized biological samples. Recovery is 95–97% for liver extracts spiked with Co-A and acetyl-CoA. Many factors may influence the tissue concentrations of CoA and acetyl-CoA (e.g., age, fed, or fasted state). Nevertheless, the values obtained by the present HPLC method for the concentration of CoA and acetyl-CoA in selected rodent tissues are in reasonable agreement with literature values. The concentrations of CoA and acetyl-CoA were found to be very low in rat plasma, but easily measurable by the present HPLC method. The method should be useful for studying cellular energy metabolism under normal and pathological conditions, and during targeted drug therapy treatment.

## 1. Introduction

Coenzyme A (CoA) and acetyl-coenzyme A (acetyl-CoA) are involved in a number of important biochemical reactions in the cell. For example, they play essential roles in epigenetics, signaling, and in the metabolism of carbohydrates, lipids, amino acids, and ketone bodies [1,2,3,4,5,6]. Cellular levels of CoA and acetyl-CoA may fluctuate significantly under conditions of fasting/feeding, in response to nutrients and hormones, during energetic stress, and/or cell growth [6,7,8]. Acetyl-CoA is a crucial metabolic intermediate, the concentration of which reflects the general energetic state of the cell. The concentration of acetyl-CoA influences the specificity/activity of multiple enzymes and contributes to the control of key cellular processes, including energy metabolism, mitosis, and autophagy [5]. Specifically, acetyl-CoA operating as a metabolic intermediate and as a second messenger determines the balance between cellular catabolism and anabolism [5]. Of particular significance, acetyl-CoA is critically important in regulating the synthesis and degradation of pyruvate [1,5]. CoA levels in tissues are tightly regulated and depend on a number of nutritional conditions that help to maintain glucose homoeostasis [1,6,7]. For example, the biosynthesis of CoA is controlled in part by the activity of the isoenzymes (isoforms) pantothenate kinase 1 and pantothenate kinase 2 (PanK1 and PanK2). Mutations of the *PanK1* and *PanK2* genes are associated with PanK-dependent neurodegeneration (PKAN) and diabetes [1,9]. It has been suggested that the development of modulators of PanK activity represent a promising approach to the treatment of both PKAN and diabetes [10]. Thus, a simple and reliable method for simultaneous measurement of CoA and acetyl-CoA in biological samples would be helpful to researchers studying diabetes and certain rare genetic neurodegenerative diseases.

Many methods have been published for the measurement of CoA and acetyl-CoA in biological samples, including radiochemical methods, spectrophotometric/fluorometric enzyme cycling methods, high-performance liquid chromatography (HPLC), high-performance capillary electrophoresis (HPCE), and mass-spectrometry (MS) [11,12,13,14,15,16,17]. Kits are also commercially available for the fluorometric determination of acetyl-CoA (e.g., Sigma-Aldrich, Saint Louis, MO, USA) and CoA (e.g., Abcam, Cambridge, MA, USA). Each method has certain advantages, but also restrictions and concerns due to the limited analysis sensitivity and/or complexity. For example, advanced LC-MS/MS techniques for the measurement of CoA and acetyl-CoA are highly sensitive [16], but also are more expensive to use. Increased costs include, but are not limited to, yearly services, maintenance, repair, and high operator salary costs. Therefore, LC-MS/MS technique is not cost effective for multiple, routine analyses under regular laboratory settings. Despite some concerns regarding the complexity of sample preparation, disposal of potentially hazardous effluents, and the possible unsuitability for high-throughput analyses, HPLC methods for determination of acetyl-CoA and CoA have proved to be useful in a variety of biological settings [17,18,19,20,21].

Shibata and co-workers have introduced an especially simple and useful HPLC-based method for the simultaneous measurement of tissue CoA and acetyl-CoA (and dephospho-CoA) [21]. The limit of detection for the method is ~10 pmol per injectate for each of the three analytes. The procedure published by Shibata and co-workers appears to be more sensitive than those of previously published HPLC methods, but is less sensitive than MS-based techniques [16,20,21]

In the present work we describe an HPLC procedure for the determination of CoA and acetyl-CoA in a variety of biological samples. We used as a starting point our previous method for the HPLC separation of tricarboxylic acid (TCA) cycle intermediates [22]. Our procedure results in faster HPLC separation and higher sensitivity than previous HPLC methods. Indeed, the sensitivity of the present procedure is comparable to that obtained by MS measurements. Moreover, it was shown that there is no co-elution or interference by other metabolites in the biological samples analyzed. To illustrate the suitability of the present HPLC-based method for CoA and acetyl-CoA analysis in biological samples, we measured the levels of these metabolites in a variety of biological samples, namely cells in culture, mouse brain cortex, as well as in rat plasma, and rat tissues (liver, kidney, and brain). The results obtained by the present procedure are in general agreement with literature values.

## 2. Materials and Methods

### 2.1. Chemicals

All solutions were prepared in Millipore water (Milli-Q system, Billerica, MA, USA). Aqueous solutions (72%) of perchloric acid (PCA) and HPLC grade acetonitrile were obtained from JT Baker (Phillipsburg, NJ, USA); the trilithium salt of acetyl-CoA, the sodium salt hydrate of CoA, sodium acetate, monosodium phosphate, 85% aqueous phosphoric acid, oxaloacetic acid, citrate synthase (from porcine heart; ammonium sulfate suspension; >1000 U/mg), acetyl phosphate, Tris HCl, and dithiothreitol (DTT) were obtained from Sigma Aldrich (Saint Louis, MO, USA); phosphotransacetylase (lyophilized preparation from *Bacillus stearothermophilus*, ~1000 U/mg solid) was obtained from Boehringer Mannheim (Ridgefield, CT, USA); Dulbecco Modified Eagle’s Medium (DMEM) was obtained from VWR Life Sciences (Radnor, PA, USA); fetal bovine serum (FBS) was obtained from Gemini Bio-Products (West Sacramento, CA, USA); and, the Bio-Rad DC™ protein assay kit was obtained from Bio-Rad (Hercules, CA, USA). Nylon membrane filters with a diameter of 47 mm and a pore size of 0.2 µm that were used for mobile phase filtering and degassing were obtained from Pall Life Science (Port Washington, NY, USA).

### 2.2. Standard Stock Solutions

Standard stock solutions of CoA and acetyl-CoA (1–10 mM) were prepared in deionized water. Sets of CoA and acetyl-CoA standards were obtained by serial dilutions of stock solutions of the standards in 5% aqueous PCA. These standard stock solutions of CoA and acetyl-CoA were stable at −80 °C for a minimum of two years.

### 2.3. HPLC System

For our most effective HPLC analysis procedure we used Waters instruments (Milford, MA, USA), 1525 binary pump, 2707 autosampler with pre-cooled platform (4 °C), and a 2489 UV/VIS detector. Data collection and data analysis were aided by Breeze2 software (Waters Corp, Milford, MA, USA) installed on a Dell computer. The wavelength for UV detection was set at 259 nm. Separation of CoA from acetyl-CoA was achieved with an ESA Inc. (now Dionex & Thermo Fisher Scientific Company, Bedford, MA, USA) RP-C18, 150 × 3 mm, 3 µm, 120 Å (PN# 70-0636) analytical column equipped with a Phenomenex Security Guard column (Torrance, CA, USA), cartridge C18, 4 × 2 mm, (PN# AJ0-4286). The columns were maintained at room temperature, the flow rate was 0.5 mL/min, and the injection volume was 30 µL. Under these conditions CoA and acetyl-CoA elute at 3.8 and 7.8 min, respectively. The HPLC run for both metabolites is completed within 12 min without any additional time for column re-equilibration between the HPLC runs.

### 2.4. Mobile Phase

To minimize the possibility of baseline drift, an isocratic mobile phase at constant room temperature was used in the present work. The mobile phase was as described by Shibata and coauthors [21], and consisted of 100 mM monosodium phosphate and 75 mM sodium acetate. The pH was adjusted to 4.6 with concentrated phosphoric acid. Acetonitrile was added to the prepared mobile phase at a ratio of 6 (acetonitrile) to 94 (mobile phase) (*v*/*v*). Prior to HPLC analysis, the freshly prepared mobile phase was filtered and degassed (see Section 2.1 for details).

### 2.5. Preparation of Biological Samples for HPLC Analysis

#### 2.5.1. Cell Cultures

CoA and acetyl-CoA concentrations were measured in cytoplasmic hybrid cells (cybrids; herein referred to as mtDNA mutant cells) harboring a patient-derived T8993G mitochondrial DNA (mtDNA) mutation, and compared with progenitor cells harboring wild type (WT) mtDNA (143B osteosarcoma cell line). The T8993G mutation, which is in subunit 6 of ATPase (ATP6), is associated with neuropathy, ataxia, and retinitis pigmentosa (NARP), or fatal childhood maternally inherited Leigh’s syndrome (MILS) [23]. The mutant cell line we used has 30% residual ATP synthesis activity and 60% residual respiration [23].

The mutant and WT cells were cultured in 10-cm Petri dishes at 37 °C, and 5% CO_2_ with 10 mL of DMEM containing 4.5 mg/mL glucose, 1 mM pyruvate, 4 mM glutamine, supplemented with 5% FBS, and 50 µg/mL of uridine. The cells were incubated for two days before the HPLC measurements, and achieved a density of approximately 1.5–1.7 million cells per Petri dish. Adherent cells were trypsinized, transferred into 15-mL centrifuge tubes, and centrifuged at 1000× *g* for 3 min at 4 °C. The sedimented cell pellet was washed once with 1 mL of phosphate buffered saline, and centrifuged at 1000× *g* for 5 min at 4 °C. The resulting pellet was resuspended in 0.3 mL of aqueous 5% PCA solution containing 50 µM DTT. The cell suspension was transferred into a 1.5-mL Eppendorf tube, incubated on ice for 10 min, vortexed several times, and centrifuged at 14,000× *g* for 10 min at 4 °C. The supernatant was removed and immediately analyzed by HPLC. The concentration of CoA and acetyl-CoA in the sample was calculated as nmol per mg of protein using the corresponding calibration curve. The pellet was resuspended in 0.3 mL of 0.1 M NaOH and used for protein measurements.

#### 2.5.2. Mouse Cerebral Cortex

CoA and acetyl-CoA were determined in cerebral cortices derived from 9–10-months-old WT and Mutant (Mut) mice. The Mut mice express a mutated form of mtDNA polymerase gamma (Polg) that results in mtDNA with a high burden of somatic mutations [24]. The Mut mice are a useful model for studies of age-related neurodegenerative diseases associated with mitochondrial dysfunction [24], such as Alzheimer disease and Parkinson disease. These mice are also a good model for studying the beneficial effects of physiological activity (exercise) in neurodegeneration, and for preclinical assessment of possible therapeutic interventions [24,25].

Both groups of mice (*n* = 6) were euthanized by cervical dislocation followed by decapitation. (The care of these experimental animals conformed to the guidelines for the ethical treatment of laboratory animals set by the McMaster University Animal Care and Use Committee and the Beth Israel Deaconess Medical Center Animal Care and Use Committee). The brains were quickly removed and each isolated brain was placed into ice-cold sterile saline, and dissected as described previously [24]. Briefly, the brain was placed into a chilled Brain Matrix, designed for freehand slicing of discrete regions of the brain (World Precision Instruments, RBMA-200C, Sarasota, FL, USA), cut into 1 mm thick coronal sections on wet ice, and then snap-frozen in liquid nitrogen. To 2–9 mg of the frozen cortex was added 200 µL of ice-cold aqueous 5% PCA, supplemented with 50 µM DTT. The mixture was then vortexed and homogenized by brief sonication for 6 s at amplitude 15%. During the sonication period, and for 10–15 min thereafter, the samples were kept on ice and periodically vortexed for 10 s every 2–3 min for maximal metabolite extraction. The homogenates were centrifuged at 14,000× *g* for 10 min at 4 °C. The protein-free supernatant was centrifuged once again under the same conditions, and a 100-µL aliquot was transferred into an HPLC vial for direct injection into the HPLC system. The concentrations of CoA and acetyl-CoA in the sample were calculated as µmol per g of wet tissue using the corresponding calibration curves.

#### 2.5.3. Rat Tissues

Four-month old female outbred Wistar rats (130–140 g weight) were euthanized by decapitation. (The care of these experimental animals conformed to the guidelines for the ethical treatment of laboratory animals set by the Institutional Review Board at the Bach Institute of Biochemistry). Tissue samples were rapidly excised and immediately frozen in liquid nitrogen. Ice-cold aqueous 5% PCA (200 µL) containing 50 µM DTT was added to the frozen brain, liver or kidney tissue sample (10–20 mg), vortexed and homogenized by brief sonication for 12 s at amplitude 20%. The rat tissues were processed as described above for mouse cerebral cortex. The concentrations of CoA and acetyl-CoA in the sample were calculated as nmol per mg of protein using the corresponding calibration curves.

#### 2.5.4. Rat Plasma

Each rat plasma sample (50 μL) was separately added to a tube containing 250 µL of ice-cold aqueous 5% PCA, supplemented with 50 µM DTT. The acid-treated extract was vortexed, centrifuged, and processed as described above. The concentrations of CoA and acetyl-CoA in the plasma samples were calculated using the corresponding calibration curves.

#### 2.5.5. Treatment of Neutralized Tissue Extracts with Citrate Synthase (CS)

In some experiments, neutralized PCA extracts were treated with CS to ensure that the HPLC peak assigned to acetyl-CoA is due entirely to acetyl-CoA, and contained no UV-absorbing compound(s) that elute(s) with the same retention time (RT) as that of acetyl-CoA [20]. CS catalyzes the following reaction (Equation (1)):

Acetyl-CoA + oxaloacetate ⇆ CoA + citrate(1)

To 50 µL of PCA-treated rat liver extract neutralized with a minimal amount of 2 M K_2_CO_3_ (final pH 7.4), followed by the removal of insoluble potassium perchlorate by centrifugation, was added 10 µL of 1 M Tris buffer (pH 7.4), 5 µL of freshly prepared 10 mM oxaloacetate, 25 µL of deionized water, and 10 µL of a solution of CS (~94 U/mL, dissolved in deionized water). The resulting reaction mixture was incubated for ~5 min at 37 °C, and a 50-µL aliquot was transferred directly into a vial for immediate HPLC-UV analysis.

#### 2.5.6. Treatment of Neutralized Tissue Extracts with Phosphotransacetylase (PTA)

In some experiments, neutralized PCA extracts were treated with PTA to ensure that the HPLC peak assigned to CoA is due entirely to CoA, and contained no UV-absorbing compound(s) that elute(s) with the same RT value as CoA [20]. PTA catalyzes the following reaction (Equation (2)):

CoA + acetyl phosphate ⇆ acetyl-CoA + P_i_ (2)

To 50 µL of PCA-treated rat liver extract, neutralized with the minimal amount of 2 M K_2_CO_3_ (pH 7.4), followed by the removal of insoluble potassium perchlorate by centrifugation, was added 20 µL of 1 M Tris buffer (pH 7.4), 20 µL of 10 mM acetyl phosphate, and 10 µL of a solution of PTA (~100 U/mL, dissolved in deionized water). The mixture was incubated for ~5 min at 37 °C and a 50-µL aliquot was transferred into a vial for immediate HPLC-UV analysis. It should be noted that although the reaction is freely reversible, the backward direction of Equation (2) is favored [26]. Thus, a large excess of acetyl phosphate over the expected concentration of CoA in the tissue sample is required to ensure complete removal of CoA. Moreover, Tris was chosen as a buffer rather than phosphate, because phosphate is a product inhibitor of the forward reaction [26].

### 2.6. Protein Analysis

For protein measurements a standard Bio-Rad DC™ protein assay kit (cat#500-0113, 500-0114, 500-1115) was used. Pellets obtained from deproteinized tissue/plasma samples were dissolved by brief 10 s vortexing in 0.5 mL of 0.1 M NaOH. For protein analysis a six fold dilution of the suspension in deionized water was used. Protein concentration was measured spectrophotometrically, using a SpectraMax M5 platereader (Molecular Devises, Sunnyvale, CA, USA) set at a wavelength of 670 nm.

### 2.7. Statistical Analysis

Data are presented as the Average ± the Standard Deviation; *p* values were determined by the two tailed paired *t* test.

## 3. Results

### 3.1. Measurement of CoA and Acetyl-CoA in Combined Standard Solutions by HPLC

**Figure 1:**
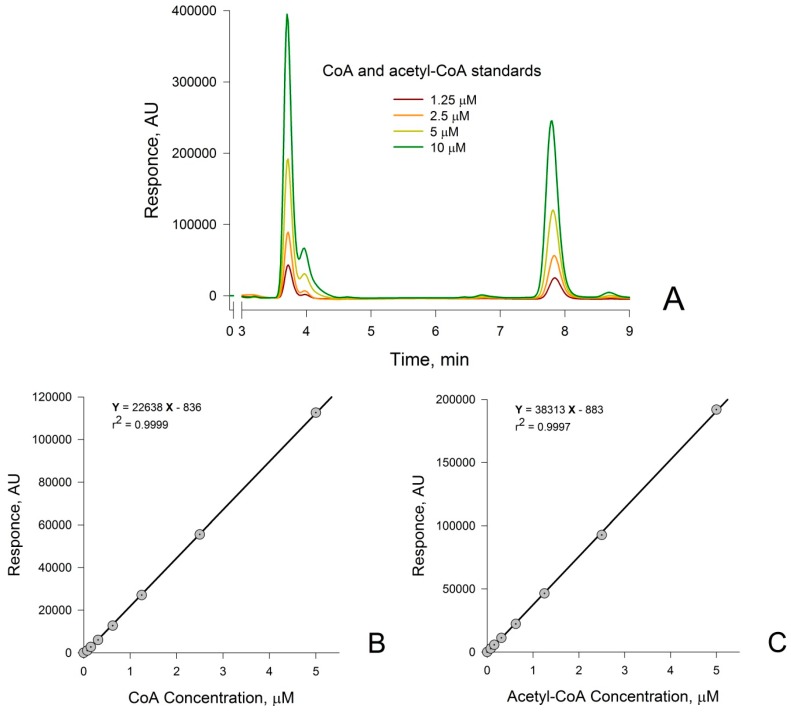
Representative chromatograms for (**A**) Coenzyme A (CoA) and acetyl-coenzyme A (acetyl-CoA) standards 1.25–10 µM (the retention times (RTs) for CoA and acetyl-CoA are 3.8 min and 7.8 min, respectively); and calibration curves for (**B**) CoA and (**C**) acetyl-CoA standards measured over the physiologically relevant concentration range of 0–5 µM. AU, arbitrary units.

Using the HPLC conditions described in the Materials and Methods section, we were able to accurately quantify CoA and acetyl-CoA in standard solutions. Representative chromatograms for combined CoA and acetyl-CoA standards are shown in Figure 1A. Calibration curves for CoA and acetyl-CoA standards were shown to be linear over the entire concentration range from 0 to 1 mM (data not shown). Only the calibration plots at the physiologically relevant range of 0–5 µM for both CoA and acetyl-CoA are shown (Figure 1B,C, respectively). Values for the peak height for each sample were plotted against known concentrations of individual standards. The equations of the linear regressions for the resulting plots (shown in panels B and C) were used to determine CoA and acetyl-CoA concentrations in the sample.

Having established that CoA and acetyl-CoA are well separated by our HPLC system, we next determined whether the procedure is applicable to biological samples. This was found to be the case. Individual concentrations of CoA and acetyl-CoA in these samples were normalized either to protein content (nmol per mg of protein) or to µmol per g of tissue wet weight. The coefficient of variation (CV, %, *n* = 3) for CoA and acetyl-CoA measurements in the standards was <1%. The CVs for CoA and acetyl-CoA measurements in the biological samples were within 1–3%. The recovery was determined by addition of known amounts of CoA and acetyl-CoA to the frozen tissue sample *simultaneously* with addition of the PCA deproteinizing agent. After extraction with the PCA reagent, and the removal of precipitated protein by centrifugation, the recovery for both metabolites was within 95–97%. The CoA and acetyl-CoA in the prepared sample extracts were stable at 4 °C for at least 24 h after preparation. The limit of detection (LOD) of our procedure is one to almost two orders of magnitude more sensitive than that described for previous HPLC methods [19,20,21]. For example, Shibata et al. [21] reported an LOD of ~10 pmol per injectate for both CoA and acetyl-CoA. By contrast, the LOD for CoA and acetyl-CoA, obtained by the present procedure are 0.0038 and 0.012 µM, respectively, or 0.114 and 0.36 pmol per injectate, respectively. The increase in sensitivity of our procedure, when compared to that of previous HPLC procedures may be due to the high sensitivity of the UV/Vis detector used in the present work.

### 3.2. Confirmation that the HPLC Peaks at 3.8 min and 7.8 min Obtained from a Rat Liver Homogenate Are Due Solely to CoA and Acetyl-CoA, Respectively

The present detection method is based on UV absorption at 259 nm. Thus, it is possible that additional compounds that also absorb at 259 nm elute from the HPLC with identical RT values to those exhibited by CoA and acetyl-CoA. Accordingly, it was necessary to determine whether the peaks obtained with RT values of 3.8 min and 7.8 min in biological samples are due exclusively to CoA and acetyl-CoA, respectively.

**Figure 2:**
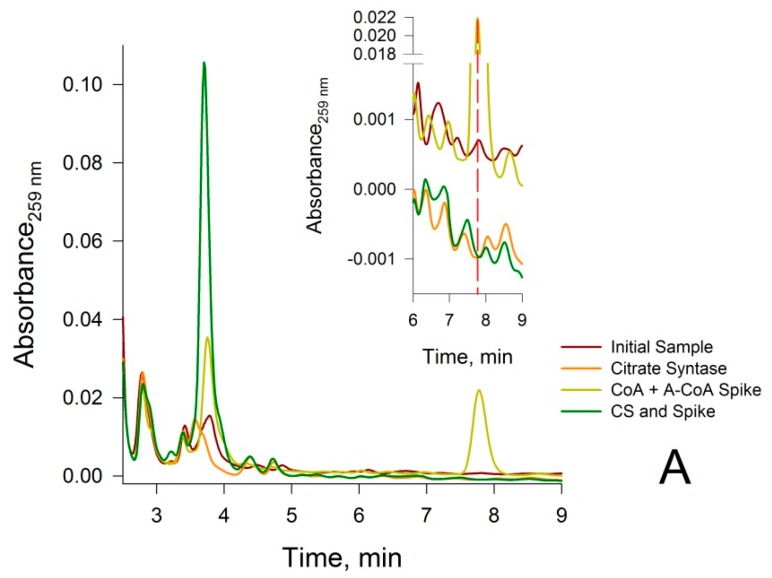

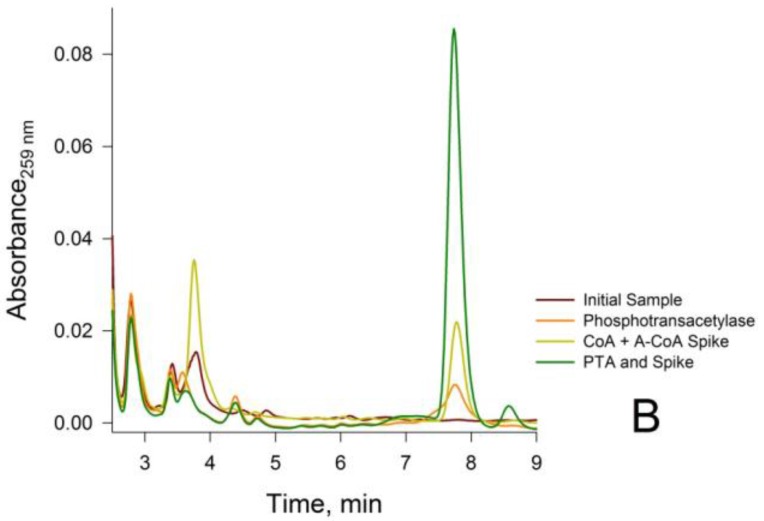
Confirmation of the identity of CoA and acetyl-CoA in the peaks eluting at 3.8 and 7.8 min, respectively, for neutralized deproteinized rat liver extracts. (**A**) Use of citrate synthase (CS) to confirm that the peak at 3.8 min is due to CoA. Brown trace: initial sample prior to treatment with CS and oxaloacetate; Orange trace: sample treated with CS and oxaloacetate for 5 min at 37 °C; Yellow trace: sample spiked with 10 µM CoA, 10 µM acetyl-CoA and oxaloacetate at zero time; Green trace: sample spiked with 10 µM CoA, 10 µM acetyl-CoA and oxaloacetate after incubation with CS for 5 min at 37 °C. Note that the magnitude of the CoA peak in the initial sample at the magnification shown is relatively small. The insert depicts an expanded scale shown for better resolution; (**B**) Use of phosphotransacetylase (PTA) to confirm that the peak at 7.8 min is due to acetyl-CoA. Brown trace: initial sample; Orange trace: sample after 5 min treatment at 37 °C with PTA and acetyl phosphate; Yellow trace: sample spiked with 10 µM CoA, 10 µM acetyl-CoA, PTA and acetyl phosphate at zero time; Green trace: spiked sample after incubation with PTA and acetyl phosphate for 5 min at 37 °C.

Shibata et al [21] previously considered the possibility that contaminants might contribute to the intensities of the peaks assigned to CoA and acetyl-CoA, respectively, in their HPLC procedure. The authors suggested that the complete disappearance of the CoA and acetyl-CoA HPLC peaks that takes place in the tissue during a 7-h incubation period at 37 °C (a process that the authors termed autocatalysis) is evidence that the HPLC peaks assigned to CoA and acetyl-CoA are not contaminated with other compounds. However, it is possible that endogenous enzymes might also catalyze the disappearance of metabolites that co-elute with CoA or acetyl-CoA. Therefore, in the current study, the identity of CoA and acetyl-CoA in biological samples was confirmed by two different procedures. In the first procedure, a deproteinized rat liver sample was spiked with authentic CoA and acetyl-CoA standards. It was shown that that the “spikes” exactly coincide with the peaks assigned to CoA and acetyl-CoA in the deproteinized tissue sample (Yellow traces in Figure 2A,B). In the second procedure, it was shown that treatment of neutralized rat liver PCA extracts with appropriate enzymes and co-substrates results in a complete disappearance of peaks at 3.8 min (assigned to CoA) and 7.8 min (assigned to acetyl-CoA) (Figure 2A,B). The enzymatic procedures were modified from those previously described by Tsuchiya et al. [20]. Before applying these assays to the biological samples, the enzymatic reactions were tested with a simulated CoA and acetyl-CoA, combined with standard mixture treated with PCA, and neutralized with 2 M K_2_CO_3_ (pH 7.4). We confirmed that treatment of the neutralized PCA extract with CS and oxaloacetate results in a complete removal of the acetyl-CoA peak, which is accompanied by a corresponding increase in the CoA peak (data not shown). We also confirmed that treatment of the neutralized PCA extract with PTA and acetyl phosphate results in removal of the CoA peak, accompanied by a corresponding increase in the acetyl-CoA peak (data not shown).

Next, a neutralized rat liver PCA extract was incubated with CS in the presence of oxaloacetate. After incubation of the extract with CS, a complete disappearance of the acetyl-CoA peak was observed together with a concomitant increase in the CoA peak (Figure 2A). In addition, incubation of the neutralized rat liver PCA extract with PTA in the presence of acetyl phosphate resulted in complete disappearance of the CoA peak, and a concomitant increase in the acetyl-CoA peak (Figure 2B). Moreover, incubation of the neutralized rat liver PCA extract (50 µL), spiked with 10 µL of 50 µM CoA or 50 μM acetyl-CoA, with either CS or PTA (and appropriate co-substrates) for 5 min at 37 °C produced similar results (Figure 2A,B). These results confirm that the peak signals in the rat liver extract at 3.8 min and 7.8 min are due solely to CoA and acetyl-CoA, respectively.

### 3.3. Determination of CoA and Acetyl-CoA Concentrations in Biological Samples

The concentrations of CoA and acetyl-CoA obtained by the present HPLC procedure for different biological samples are shown in Table 1.

## 4. Discussion

### 4.1. WT and mtDNA Mutant Cells

As noted in the Material and Methods section, mtDNA Mut cells exhibit decreased ATP synthesis and respiration. We hypothesized that as a result of this severely compromised energy deficit the concentrations of CoA and acetyl-CoA would also be severely compromised in the Mut cells. This hypothesis was shown to be correct (Table 1). The concentrations of CoA and acetyl-CoA in the cell extracts were measured (*n* = 6 for each group). Representative averages of experiments are shown in Table 1. In all cases, the concentrations of CoA were found to be at least ten fold greater in the WT cells than in the Mut cells (Table 1; *p* < 0.001). The concentration of acetyl-CoA in the Mut cells was also lower than that in the WT cells. However, in this case the difference in the concentration of acetyl-CoA between these two cell lines was only about 15–20%, but the difference is still highly significant (Table 1; *p* < 0.005).

### 4.2. Rat Liver and Kidney

We have compared the concentration values for CoA and acetyl-CoA in rat liver and kidney obtained by the present procedure to representative literature values (Table 2, [21,27]). Note that methods to measure CoA and acetyl-CoA compiled by Bergmeyer [27] predate 1974—a time when metabolites were often measured by enzymatic procedures. Indeed, measurements of acetyl-CoA and CoA during this period were commonly carried out either by direct spectrophotometric measurement of an enzyme product, or by an amplification enzyme cycling method [6,13,28]. It is interesting to note that these methods yielded values of mammalian liver CoA and acetyl-CoA concentrations that are very similar to values obtained by modern HPLC procedures (discussed further below). Note also that in Table 1 we report these concentrations in terms of nmol/mg of protein. However, most concentrations of CoA and acetyl-CoA reported in the literature are in units of μmol/g wet weight (or nmol/g wet weight). Thus, assuming that 1 g wet weight of tissue contains 100 mg protein [29], then the concentrations of CoA and acetyl-CoA in rat liver (from Table 1) are ~87 and ~19 nmol/g wet weight. These values are in good agreement with representative, previously published data for rat liver (Table 2). The concentrations of CoA and acetyl-CoA have also been measured in mouse liver. The concentrations were reported to be ~160 and 105 nmol/g wet weight, respectively [30]. Thus, the concentration of CoA in mouse liver is similar to that noted by several authors for rat liver (see Table 2). However, the concentration acetyl-CoA is considerably higher in mouse liver than that reported for rat liver (Table 2). This may be due in part to species differences. The concentration of acetyl-CoA in cow liver has been reported to be ~34 nmol/g wet weight [31]. The concentrations of acetyl-CoA and CoA in sheep liver have been reported to be ~50 nmol/g wet weight [28]. In conclusion, the concentration of hepatic CoA seems to be remarkably constant among the four mammalian species (~50–160 nmol/g wet weight). The concentration of hepatic acetyl-CoA among these species is somewhat more variable (19–105 nmol/g wet weight).

By using the same assumption that 1 g wet weight of tissue contains 100 mg of protein, the average concentrations of CoA and acetyl-CoA in rat kidney (from Table 1) obtained by the present procedure are ~19 and 1.3 nmol/g wet weight (Table 2). These concentrations are somewhat lower than those reported by Shibata et al. [21] and by Bergmeyer [27]. This variability may be due in part to the nutritional state of the experimental animal, whether or not the experimental animal was exposed to an anesthetic, and/or time between sacrifice and tissue extraction. In this context, we note that acetyl-CoA concentrations have been reported to be 2–6 fold higher in the livers of 24–48 h starved rats than in the livers of fed rats [27], but others have found no change in the concentration of rat liver acetyl-CoA after 24 h starvation [32].

### 4.3. Rat Plasma

We were unable to find values for plasma concentrations of CoA and acetyl-CoA in the scientific literature. The concentration of CoA (~9 nM) and acetyl CoA (~0.3 nM) in the plasma of female Wistar rats (4 month old; 130–140 g) (Table 1) is relatively low, emphasizing the sensitivity of the present HPLC method. The present work apparently provides the first estimates of the concentration of both CoA and acetyl-CoA in plasma. It is suggested that the procedure will be useful in correlating the plasma concentration of these compounds with various disease states.

### 4.4. Rodent Brain

Measurement of metabolites in the rodent brain is challenging. During the time it takes to remove the brain from the skull, the tissue will be subjected to anoxia/ischemia which may dramatically affect metabolite concentrations, especially metabolites (including energy metabolites) that turn over rapidly. For example, more than 50 years ago Lowry et al. [33] showed that within tens of seconds following decapitation there is substantial loss of glucose, and a considerable increase in lactate in mouse brain. Since the production of acetyl-CoA and CoA are intimately associated with energy metabolism it is possible that these metabolites will also be altered in the ischemic brain. Kato [34] noted a slight decrease in the concentration of whole mouse brain acetyl-CoA within 10–20 s of decapitation, followed by a return to baseline levels by 30 s. In the study of McDougal and Dargar the concentration of acetyl-CoA in mouse cerebral cortex decreased slightly after one minute of anoxia/ischemia [14]. However, in later work Říčný and Tuček [11] investigated the effect of short periods of anoxia/ischemia on the concentration of acetyl-CoA in brain tissue from 3-day-old rats, and found that following decapitation the concentration of cerebral acetyl-CoA increased within a minute by about 2.7 fold, when compared to that obtained for brain “fixed” almost immediately in live rats by microwave irradiation [11]. The baseline concentration of acetyl-CoA obtained by Říčný and Tuček for rat brain fixed by microwave irradiation was found to be ~1.9 nmol/g wet weight [11]. This value is lower than that obtained by Allred and Guy [13] for a whole brain of ~8 nmol/g wet weight for adult, fed rats (however, these authors did not specify how the brain was extracted.) In the present work we noted a concentration of acetyl-CoA in the whole rat brain of ~0.028 nmol/mg protein (Table 1). Assuming that 1 g of brain contains 100 mg protein [29,35], then the concentrations of CoA and acetyl-CoA in rat brain by the present HPLC method are ~8 and ~2.8 nmol/g wet weight, respectively. The present value for the cerebral concentration of CoA (~8 nmol/g wet weight) in adult rat brain is within the range (~23 nmol/g wet weight) noted by Allred and Guy for young rats [13]. The present value for the cerebral concentration of acetyl-CoA in rat brain (~2.8 nmol/g wet weight) is within the range noted above as reported by Říčný and Tuček [11], and Allred and Guy [13] of ~1.9 and ~8 nmol/g wet weight, respectively.

We are aware that our procedure for measuring CoA and acetyl-CoA in rat brain involves a step that subjects the brain to a short period of anoxia/ischemia. Thus, the present values for the concentration of CoA and acetyl-CoA in rat brain are tentative. Nevertheless, the values are in reasonable agreement with literature values, and suggest that our present method would be useful as a general procedure for measuring CoA and acetyl-CoA in rodent brain.

In addition to determining CoA and acetyl-CoA in rat brain, we determined the concentration of these metabolites in mouse cortex, obtained from both WT and Mut mice. As noted above, the Mut mice express a mutated form of mtDNA Polg that results in mtDNA with a higher burden of somatic mutations. These damaged mitochondria are likely to be less effective in energy metabolism. As shown in Table 1, the concentrations of both CoA and acetyl-CoA in cerebral cortex were found to be somewhat lower in Mut mice when compared to those of WT mice. The observed trend in the differences (~20% and 40% decrease in CoA and acetyl-CoA concentrations, respectively) is not statistically significant due to a very high biological variability in mice compared to those noted for WT and Mut cells in culture, where the data are much less variable (*p* < 0.001 and *p* < 0.005 for CoA and acetyl-CoA, respectively) (Table 1). Again, we acknowledge the possible pitfalls in measuring CoA and acetyl-CoA concentrations in the cerebral cortex obtained from ischemic/anoxic mouse brain. Nevertheless, the data suggests that there is a trend toward lower values for the concentrations of CoA and acetyl-CoA in the cerebral cortex of the Mut mice when compared to those in the cerebral cortex of the WT mice. Note that the concentrations of CoA and acetyl-CoA in tissues depends on a number of factors, such as how the rodent was sacrificed, the diet, fed or fasted state, and possibly, whether or not the experimental animal was exposed to anesthesia (and what type of anesthesia). Moreover, the presence/absence of mitochondrial mutations, the activity of PanK isoforms, and their localization in different cell compartments and organs are also expected to contribute to the differences in tissue concentrations of CoA and acetyl-CoA. As noted in the Introduction, PanK is an important factor in tissue-selective CoA distribution [1,2,3,4,5,6,7,8,9,36,37,38].

## 5. Conclusions

The present work describes a rapid HPLC method for the determination of CoA and acetyl-CoA in biological samples that is simpler and at least ten-fold more sensitive than are previously described HPLC procedures. The current HPLC procedure was shown to be sensitive enough to provide reliable estimates of the concentrations of CoA and acetyl-CoA in rat plasma despite concentrations in the nM to sub nM range. Moreover, the present procedure allowed us to determine the concentration of these biochemicals in very small samples (2–9 mg wet weight) of mouse brain cortex. To illustrate the general usefulness of the procedure, we measured CoA and acetyl-CoA in WT osteosarcoma 143B cells, and in the corresponding Mut rho0 cells. As expected, the concentrations of CoA and acetyl-CoA were found to be significantly lower in the Mut rho0 cells. A similar observation was made for the concentration of CoA and acetyl-CoA in the cerebral cortex of WT vs. Mut mtDNA Polg mice. The concentration of these metabolites in normal rat liver, kidney, and brain, obtained by the present HPLC procedure are in reasonable agreement with values in the literature.

It is expected that the present procedure will be useful for determining concentrations of CoA and acetyl-CoA in a variety of biological materials and in tissues of animal models of human disease.

## Figures and Tables

**Table 1:** Concentrations of CoA and acetyl-CoA in different biological samples **^a^**.

Biological Sample	N	CoA	Acetyl-CoA
WT cells ^b^	6	0.467 ± 0.015	0.162 ± 0.004
Mut cells ^b^	6	0.040 ± 0.001 ^e^	0.140 ± 0.009 ^f^
WT mouse cortex ^c^	6	0.017 ± 0.006	0.008 ± 0.003
Mut mouse cortex ^c^	6	0.012 ± 0.004	0.005 ± 0.001
Rat liver ^b^	16	0.872 ± 0.122	0.194 ± 0.038
Rat kidney ^b^	16	0.191 ± 0.062	0.013 ± 0.006
Rat brain ^b^	16	0.079 ± 0.024	0.028 ± 0.011
Rat plasma ^d^	16	0.009 ± 0.001	0.0003 ± 0.0001

^a^ Data are Average ± Standard Deviation. The concentrations are expressed in: ^b^ nmol/mg protein, ^c^ µmol/g wet tissue, ^d^ μM. ^e^ Different from the value obtained for the WT cells with *p* < 0.001. ^f^ Different from the value obtained for the WT cells with *p* < 0.005. WT, wild type; Mut, mtDNA mutant.

**Table 2:** Comparison of CoA and acetyl-CoA concentrations (nmol/g wet weight) in rat liver and kidney from various literature sources.

Bergmeyer [27] ^a^			Shibata et al. [21] N = 5		Present Findings ^b^ N = 16	
	Liver	Kidney	Liver	Kidney	Liver	Kidney
CoA	135–158	68	161	68	87.2 ± 12.2 (63–124)	19.1 ± 6.2 (8–31)
Acetyl-CoA	22–47	18–43	9	5	19.4 ± 3.8 (10–35)	1.30 ± 0.60 (0.2–3.6)

**^a^** The data are a compilation of several references. See Bergmeyer [27] for the original references. **^b^** The average values are from Table 1 assuming that 1 g wet weight of tissue contains 100 mg of protein. The numbers in parentheses represent the range of values from minimum to maximum.
